# Influence of Physical Activity on Pain, Depression and Quality of Life of Patients in Palliative Care: A Proof-of-Concept Study

**DOI:** 10.3390/jcm10051012

**Published:** 2021-03-02

**Authors:** Dariusz Myrcik, Wojciech Statowski, Magdalena Trzepizur, Antonella Paladini, Oscar Corli, Giustino Varrassi

**Affiliations:** 1 Emergency Medicine, Department of Internal Medicine, Faculty of Health Sciences in Bytom, Medical University of Silesia in Katowice, Piekarska 18, 42-600 Bytom, Poland; dariuszmyrcik@me.com (D.M.); magdalenatrzepizur@icloud.com (M.T.); 2 Chair and Department of Medical and Molecular Biology, Faculty of Medical Sciences in Zabrze, Medical University of Silesia in Katowice, Jordana 19, 41-808 Zabrze, Poland; statowski@wp.pl; 3 Faculty of Health Sciences, Jan Długosz University in Częstochowa, Armii Krajowej 13/15, 42-200 Częstochowa, Poland; 4 Department of MESVA, University of L’Aquila, 67100 L’Aquila, Italy; antopaladini@gmail.com; 5 Mario Negri Institute for Pharmacological Research IRCCS, 20156 Milano, Italy; oscar.corli@marionegri.it; 6 Paolo Procacci Foundation, Via Tacito 7, 00193 Roma, Italy

**Keywords:** pain, palliative care, mobility programs, physiotherapy, physical exercises, quality of life

## Abstract

Introduction: Palliative care not only focuses on physical ailments associated with the disease, but also considers the psychological, social and spiritual needs of the patients. The aim of this study is to assess the impact of physical activity on palliative care patients, with special regard to the subjective assessment of severity of total pain and quality of life. Materials and methods: The study was conducted on 92 palliative care patients either in a hospice or at home. The tool used to assess the patients was an original questionnaire focusing on the area of their independence and motor abilities. The study attempted to understand whether an appropriate physical activity and the instruction of palliative care patients and their families in the field of independence would improve the quality of life and reduce the intensity of total pain in the patients. Results: All of the patients were at an advanced stage of cancer. The survey at time “0”, conducted before the start of the instructions for patients and their relatives, showed that a majority of patients (47, 51.09%) often experienced limitations during the performance of daily activities. In the fourth visit, conducted one week after the fourth educational session, there was a significant increase in patients who did not experience any limitations in performing their daily activities or experienced them just sometimes. Conclusions: The ultimate effect of the proposed educational program on physical activity was an increase in the quality of life, a reduction in pain and a mood improvement. These results would need confirmation with more extensive studies.

## 1. Introduction

The World Health Organization (WHO) defines quality of life as “an individual’s perception of their position in life in the context of the culture and value systems in which they live and in relation to their goals, expectations, standards and concerns” [1]. In patients, quality of life is usually health-related, and it is treated as a dependent variable, conditioned by the disease process and the current or proposed treatment method. Health-related quality of life includes six dimensions: physical, psychological, independence, social relations, environment and personal beliefs [1,2,3]. When assessing quality of life, it is crucial to distinguish the objective health condition from the subjective feeling of the patient. Two patients affected by the same disease can assess their quality of life in very different ways. In fact, the current situation is assessed in relation to the expected individual standard of living based on, among other factors, their own experience and accepted values [4,5].

Palliative care, as defined in 2002 by the World Health Organization (WHO), is “an action aimed at improving the quality of life of patients and their families who face problems related to a life-threatening disease by means of prevention of suffering and its alleviation through early detection, thorough assessment and treatment of pain and other physical, psychosocial and spiritual problems” [4,6,7]. Currently, the intervention standard is represented by interdisciplinary teams that provide multi-directional care to terminal patients. In addition to doctors and nurses, they also include psychologists, physiotherapists, dieticians, priests and social workers. Only properly integrated action can result in effective therapies, and thus improve the quality of life of terminal patients [7,8].

Patients in palliative care often experience physical and functional impairments connected with the severity of the disease. Patients diagnosed with a hopeless neoplastic disease and other non-neoplastic diagnoses suffer from a huge burden of symptoms, such as dyspnea, lack of energy or weakness. Along with the development of the disease, the levels of physical fitness and mobility decrease and, moreover, the ability to perform everyday activities also worsens. Patients sometimes become completely dependent on their caregivers for everyday activities. Autonomous mobility is an important component of quality of life. An independent patient is able to accept the disease to a greater extent and adapt better to life in changed conditions. Thus, the physiotherapist’s role in the interdisciplinary palliative care team is significant, and the purpose of rehabilitation should be to maximize the patient’s self-reliance and independence as much as possible, regardless of the prognosis of the primary disease [2,4,5,9].

Pain is one of the most common problems found in patients in palliative care and occurs in approximately 50–80% of patients in the initial stages of treatment [10,11]. In general, at the moment of starting palliative care, the loss of mobility is often responsible for a reduction in the patient’s independence. Sometimes patients are completely dependent on other people. This may increase the severity of total pain and would become an indication to strengthen the treatments.

The aim of this study was to assess the impact of a physical activity program on pain, quality of life and functional condition of patients involved in a palliative care program. This will provide data for the literature, which at the moment are very limited, on a topic of important clinical significance.

## 2. Materials and Methods

The study was conducted on 100 consecutive patients, admitted into palliative care between December 2018 and June 2019, and managed in a hospice or at home. The main pathologies of the examined group were lung, breast and stomach cancer. All of them were in progressive disease condition. The study was part of the normal routine treatment and did not require the use of drugs. All the procedures carried out during the research, because they involved human participants, were respectful of the norms of the institutional and/or national ethics committees, as well as the Declaration of Helsinki (1964) and its subsequent amendments. All the participants were carefully informed of the protocol and signed a consent form, also for publication of their data.

The study was part of an educational program aimed at instructing patients and their caregivers in the field of physical activity and independence. It attempted to improve the patients’ quality of life and reduce the intensity of their total pain. During the study period, none of the patients was prescribed extra drugs, apart from the ones already in use.

The criterion for inclusion in the study was a Karnofsky score over 20%, obtained during the first visit. People in an agonic state were excluded. Participation in the study was voluntary and anonymous. Patients subjected to the study declared a stable level of pain intensity, which was pharmacologically treated according to the WHO analgesic ladder. The study consisted of a baseline visit and 4 subsequent visits investigating the development of the educational program.

The interdisciplinary health team participating in the implementation of the program included: a doctor, a nurse, a physiotherapist and a psychologist. At baseline, patients’ mobility, pain intensity, quality of life and depression were assessed, by means of an original questionnaire filled out by the patients themselves or with the help of the nursing staff (Supplementary Material M1). The questionnaire included items about patients’ activity and independence during everyday activities, such as moving, eating and performing personal hygiene. Independence was assessed by using a four-point scale, highlighting the necessity of help from third parties (family members or caregivers) during these activities. The questionnaire was previously studied for its scientific consistency (Supplementary Material M2). Pain intensity was measured with the numeric rating scale (NRS). Patients also rated their level of depression using Beck’s questionnaire. For each item, the answer was given on a four-point Likert scale (rarely, sometimes, often, almost always) scored increasingly from 0 to 3 points. The level of intensity of depression symptoms was calculated from the total number of points. Quality of life was measured by a five-point verbal scale (very bad, bad, difficult to say, good, very good).

Subsequently, a series of weekly educational sections on physical activity between physiotherapist, psychologist and patients and their relatives were scheduled. After each visit, patients completed the questionnaire focusing on their activities during the day and their independence in carrying out daily activities. Patients also rated the improvement in their fitness over the past week.

This program was repeated 5 times for 5 consecutive weeks. Finally, the patients were requested to assess again all the baseline parameters, namely, activity levels, pain intensity, quality of life and depression status. The topics of educational visits included:

Visit 1:

- Independence of the patient
- Mobilization of the patient, so as to be independent
- Education of the patient and the caregiver in the field of physical adaptation techniques
  ○ Self-movement
  ○ Training within the immediate environment
  ○ Independence in eating
  ○ Independence in using the toilet
  ○ Use of the telephone
  ○ Use of multimedia receivers

Visit 2:

- Safeguarding techniques used to minimize the likelihood of falls, such as:
  ○ Adapting the apartment and the immediate environment for the safe movement of the palliative person
  ○ Safe falling techniques
- Use of rehabilitation equipment and orthopedic supplies in safeguarding patients
  ○ Education of the patient in the field of walking with a support
  ○ Education of the patient in the field of moving around in a wheelchair, including overcoming architectural barriers
  ○ Use of mechanical equipment for patient transfer
  ○ Education on how to equip the dwelling with handrails, railings, handles, non-slip mats, shower chairs etc.
  ○ Education in the field of equipping the patient with the necessary aids to perform basic and complex everyday activities, as well as activities related to leisure time and rest
- Relaxation techniques
  ○ Education with basic elements of autogenic training and yoga

Visit 3:

- Shaping the environment of palliative patients
- Overcoming architectural barriers
- Counseling in the field of medical and rehabilitation services in mobilization of patients

Visit 4:

- Creating conditions for independent physical rehabilitation
  ○ Education in basic respiratory exercises, and stretching exercises depending on the patient’s current condition
  ○ Education in creating a home rehabilitation space
- Psychological support in overcoming their own and external limitations
- Organization of free time of a palliative patient

Visit 5:

- Integration with the environment and its impact on the quality of life of palliative care patients
- Adaptation of a palliative patient to function in changing life conditions
- Quality of life and acceptance of the disease

### Statistical Analysis

The results were collected and analyzed using a Microsoft Excel spreadsheet. Descriptive statistics were formulated. In addition, numerical and percentage lists of the examined variables were made. The results are shown as tables and figures. In order to test the hypothesis that a series of educational visits had a statistically significant effect on the physical activity and autonomy of the patients, the McNemar test, with Yates’ correction, for paired nominal data was performed, using IBM SPSS Statistics, Version 26. In view of this, a number of pairwise comparisons of each measurement with each other were made. It is reasonable to apply a correction to reduce the risk of making a Type I error. The Bonferroni correction was applied in this case.

## 3. Results

Only 92 patients out of the 100 that were screened took part in the study; 8 patients chose not to participate. The research group consisted of 35 men (38.04%) and 57 women (61.96%). Their characteristics are summarized in Table 1 and Table 2.

**Figure 1:**
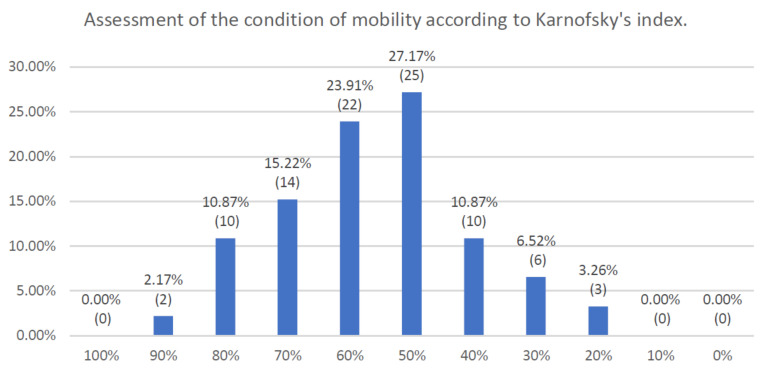
Assessment of the condition of mobility according to Karnofsky’s index.

Figure 1 shows the assessment of the general condition according to Karnofsky’s index, which had an average value of 50%, indicating a condition requiring frequent care and medical interventions. There were 25 patients (27.2%) in this condition and 22 (23.9%) with 60% Karnofsky level, indicating a condition requiring periodic care, with the ability to meet most everyday needs on their own. The two extremes of the distribution curve were represented by 2 patients with an index equal to 90%, with quite a normal level of activity, and 3 patients with a 20% index, in very serious clinical conditions, with a need to recover in a hospice and receive supportive treatment.

**Figure 2:**
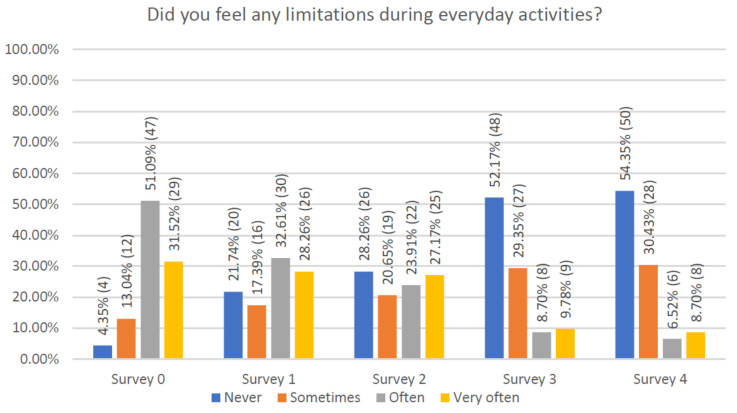
Assessment of limitations during performance of everyday activities.

The changes in limitation in performance of everyday activities, during the observation period, are shown in Figure 2. The baseline visit showed that 47 patients (51.1%) experienced frequent limitations during daily activities. At the last visit, more than 50% of patients declared that they had no limitations. Throughout the follow-up, there was a constant improvement in activity as the educational program progressed.

**Figure 3:**
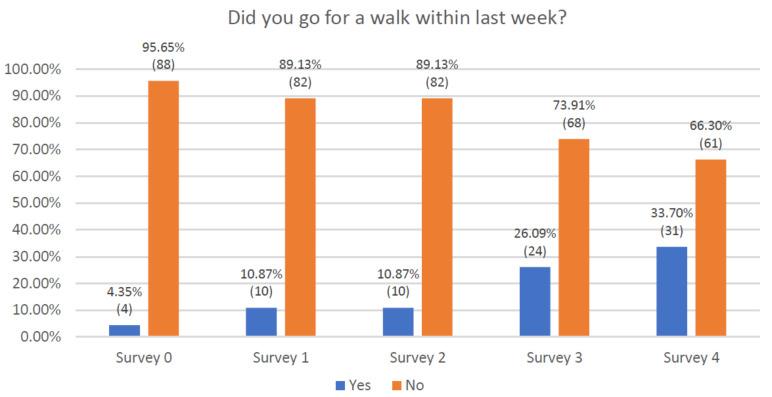
Declaration of respondents regarding participation in a walk.

Patients were also asked about participation in a walk each week (Figure 3). Before starting the educational sessions, only 4 (4.35%) patients reported they had a short walk in the last 7 days. At the last visit, 31 patients (33.7%) were able to walk at least 5 consecutive minutes during the previous week. The statistical analysis of the data showed a significant increase in the performances of the patients (Tables S1–S6).

**Figure 4:**
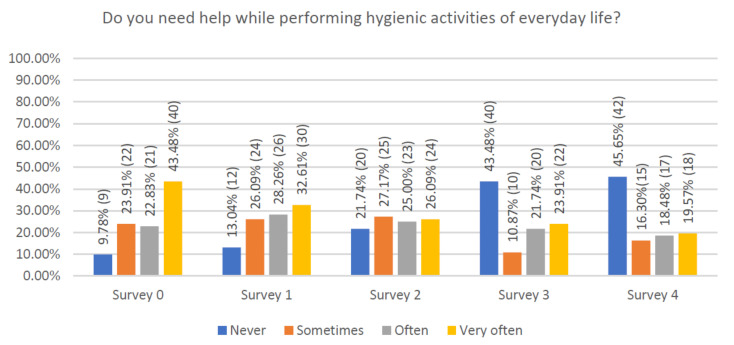
Declaration of respondents regarding help during everyday hygienic activities.

The majority of participants in the study, before the training program, often or very often required help in performing everyday personal hygiene (Figure 4). In contrast, fully autonomous subjects initially amounted to about 10% and their percentage rose to more than 40% by the end of the study.

**Figure 5:**
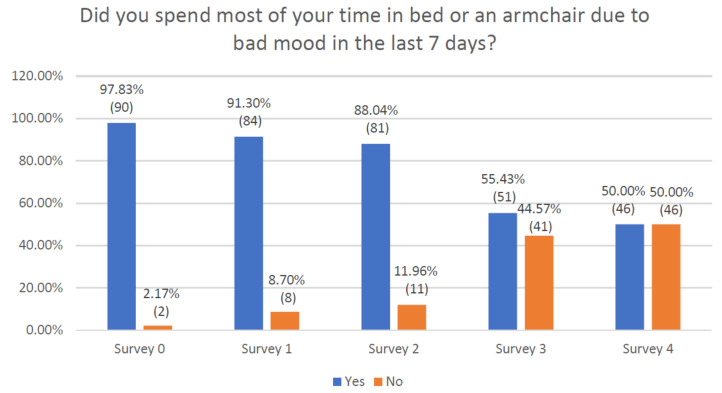
Declaration of respondents regarding passive spending of time resulting from a bad mood.

The following question was “Did you spent most of your time in bed or an armchair due to bad mood in the last 7 days?”. The answer was positive in 97.3% of patients at baseline visit, and progressively decreased to 50% by the end of the observation. (Figure 5). The statistical analysis of these data also showed significant results (Table S7).

**Figure 6:**
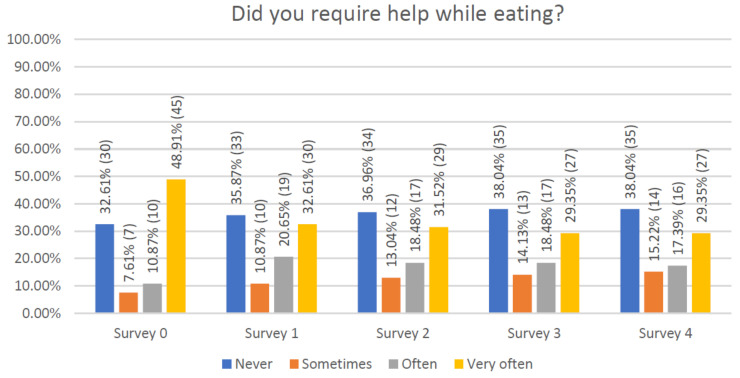
Declaration of respondents regarding independence while eating meals.

A similar question was asked about the need for help while eating meals (Figure 6). Initially, this requirement stood at 48% but then fell and remained stable at around 30%.

**Figure 7:**
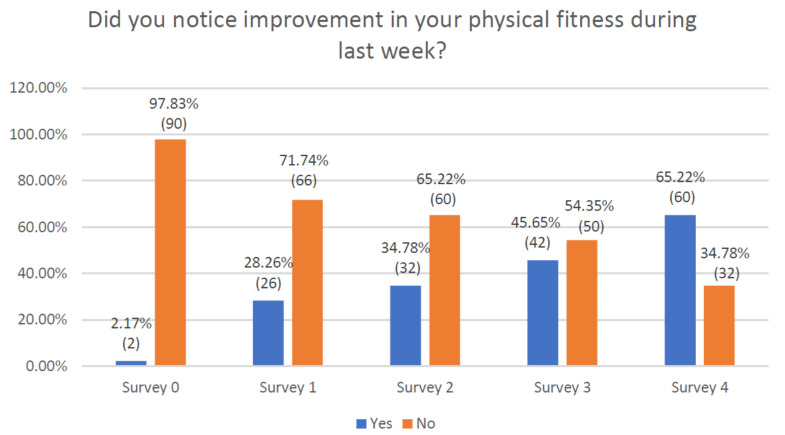
Declaration of respondents regarding improvement in their fitness over the last week.

Evaluating the overall improvement in physical fitness, week after week, patients expressed an initial negative impression in 97.8% of cases, to reach a value of less than 40% by the end of the study (Figure 7). The statistical analysis of the data showed a progressive significant increase in the weekly physical fitness. The differences were not significant in the comparisons between the last two weeks (Table S8).

**Figure 8:**
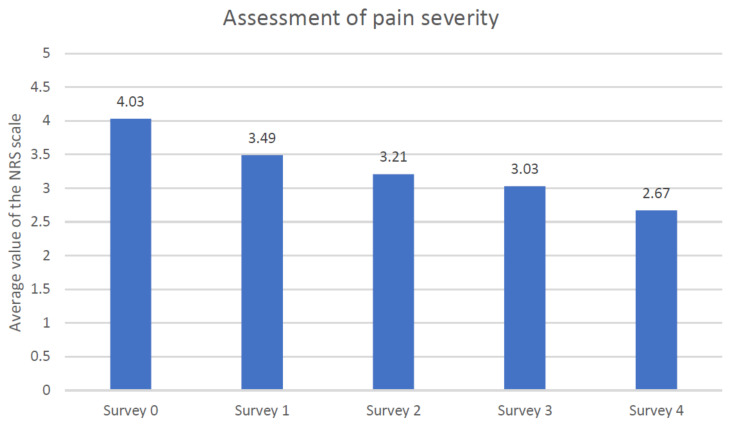
Pain severity assessment using the numeric rating scale (NRS).

**Figure 9:**
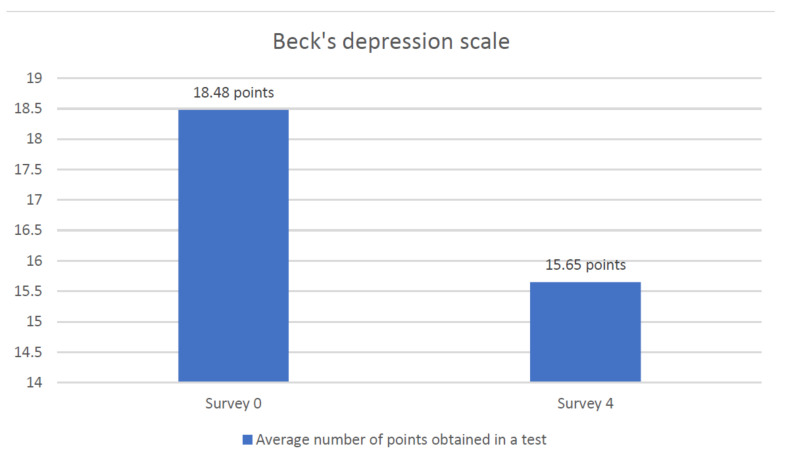
Assessment of the level of depression of respondents using Beck’s depression scale.

All the data just introduced go univocally in the direction of an improvement in activities and autonomy. These results were, in the project of this study, the premise to assess how other symptoms (pain and depression) and quality of life of patients could change. Pain, based on the NRS scale, decreased from 4.03 to 2.7 from the first to the last visit (Figure 8). The level of depression, using Beck’s depression scale, was 18.5 at the first visit and 15.6 after the last educational session (Figure 9).

**Figure 10:**
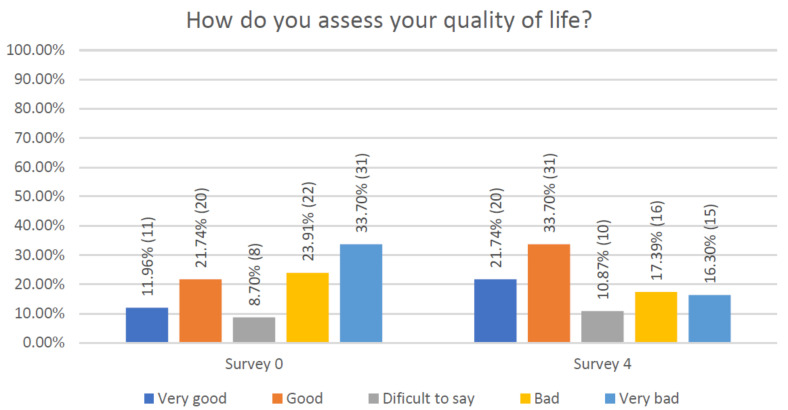
Quality of respondents’ life including the assessment of pain and mood.

As for the quality of life, at the first visit patients graded it as “very bad” or “bad”, respectively, in 33.70% and 23.91% of cases, while 12% indicated a “very good” quality of life. At the completion of the entire educational program, the negative evaluations became, respectively, 16.3% and 17.4% (Figure 10).

**Figure 11:**
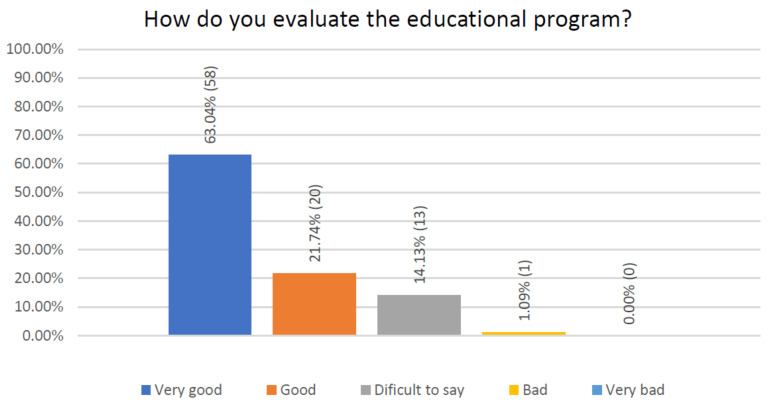
Satisfaction assessment after coordination of the educational prescribed program.

When assessing the level of patient satisfaction after coordinating the educational program, patients mostly evaluated and accepted it very well (Figure 11). Only 1.09% of the respondents declared a bad rating.

## 4. Discussion

This study has clearly shown that physical education programs help in increasing the quality of life of palliative care patients. In recent years, quality of life has received growing attention in research [12,13], including in palliative care settings. Palliative care is not only medical help for terminally ill patients but also provides motivated interventions for the control of suffering and symptoms related to chronic progressive diseases, trying to improve, or at least to maintain, an acceptable quality of life. Improvement of the quality of life of palliative patients is a huge challenge for health care professionals. Frequently, pharmacological treatment, even if prescribed on a rational basis, [14] is not enough. Physical and psychological indices are very important to manage pain [15], and when a peripheral neuropathy is present, non-pharmacological management may be helpful [16].

Most patients prefer to spend the period of illness at home. Patients, after diagnosis, lose their desire to participate in social life. Their exhaustion from therapeutic interventions is additionally intensified, and their quality of life is significantly reduced, and this frequently occurs because of chronic pain [17]. Support from health staff working with the patient and the family is an important tool. The assistance of a psychologist and the ability to cope with a traumatic stressor are important components in care for quality of life [18], as also shown by our study.

Often, relatives do not have any knowledge about care and treatment of a palliative patient. Problems associated with a chronic disease, such as cancer, affect all levels of life and functioning of the whole family. Each family member may react in a different way to the stress related to the disease. Some are ready to fight and help the sick person, while others prefer to avoid confrontation with reality. Commonly, friends/relatives of the affected individual often think that, if the patient is ill, he or she should not do anything, and that the interdisciplinary health team should fully take control of the patient, including support for most of the basic activities. The purpose of the training program applied in this study was to educate patients and their relatives in the field of independence and activity. The number of studies related to physiotherapy in terminal patients is increasing. The main goal of rehabilitation in palliative medicine is to improve quality of life by alleviating symptoms and optimizing the level of physical fitness in daily activities [19]. This study followed certain methodological steps: proposing a training program aimed at improving physical activities and patient autonomy, and evaluating the results obtained step by step. At the same time, it assessed the correlations with the presence and intensity of pain, depression level and quality of life. In all cases, the improvement in physical performance went in parallel with the three parameters examined. In other terms, if exercise is properly taught and monitored, then the outcomes for palliative care patients are significantly better.

A recent systematic review focused on the effects of physical activity in cancer prevention and survival [20]. Other previous studies show that exercise conducted in patients diagnosed with cancer reduces anxiety, stress and depression, also reducing the levels of pain, fatigue, breathlessness and insomnia [21,22]. Pyszora et al. [23] developed a rehabilitation program aimed at cancer patients and showed an improvement in general well-being and a reduction in the severity of co-occurring symptoms, especially pain, drowsiness, loss of appetite and depression. In other terms, the interplay between cancer and physical activity is increasingly being studied. Much less work has been done in palliative care. The program we proposed was positively evaluated by patients who completed a treatment satisfaction questionnaire, as shown in the results. Similar results were obtained by others, stating that rehabilitation contributes to better physical functioning and subjective improvement of well-being of patients in palliative care, including a significant impact on reduction of pain and mood improvement [24].

### Limitations and Strengths

This is a simple pilot research study aiming to find the best methodology for an optimal treatment in palliative care patients. The objective was to increase their quality of life, using an educational program for physical activity that could increase their autonomous activities. Now that we have a better understanding, this issue is not only to be studied on a theoretical basis but will likely become the topic for a larger study that will include more patients in a randomized clinical trial setting, in order to obtain highly valuable scientific data. In any case, the tools adopted in this study are good enough to support future studies. Moreover, a strong point of our research is its practical dimension. The study may find applications in other medical specialties, e.g., in geriatrics or long-term care. The educational tools used during the pilot study will be subject to further observation in subsequent studies and (after a potential correction) will be able to serve as a permanent therapeutic tool supporting the daily practice of treating palliative care patients.

## 5. Conclusions

1. Palliative patients often or very often experience limitations in the performance of daily activities.
2. The examined group of respondents in the majority of cases lead a limited lifestyle and report problems in the sphere of independence, even performing basic activities of everyday life.
3. After completing the educational program for a useful physical activity, the majority of patients declared a reduction of limitations in routine everyday activities.
4. The derived effect was an increase in the quality of life of the examined group of palliative patients, including reduction of pain and mood improvement.
5. The original educational program for physical activity generated a positive response among patients in palliative care, both in a hospice or at home, and their caregivers.
6. The implementation of this project in the everyday management of palliative care patients would need little additional efforts, in terms of personnel, and would provide good results, if those obtained with this pilot study are confirmed by further studies.

## Figures and Tables

**Table 1:** Descriptive statistics of age and morphological parameters of patients. BMI = Body Mass Index

Variable	Average	Minimum	Maximum	SD
Age	66.47	41.00	90.00	9.55
Body Mass	57.05	40.00	90.00	12.72
Height	162.57	148.00	186.00	9.73
BMI	21.50	15.63	35.16	3.87

**Table 2:** Main patient pathologies.

Men			Women		
Pathology	*N*	%	Pathology	*N*	%
Malignant Cancer of Bronchi and Lung	12	13.04%	Malignant Cancer of Bronchi and Lung	19	20.65%
Stomach Cancer	9	9.78%	Breast Cancer	16	17.39%
Prostate Cancer	6	6.52%	Colon Cancer	9	9.78%
Colon Cancer	3	3.26%	Cancer of Liver and Bile Duct	5	5.43%

## Data Availability

All data generated or analyzed during the study are presented in this published article. All the original data may be available, on reasonable request.

## Supplementary Materials

The following are available online at https://www.mdpi.com/2077-0383/10/5/1012/s1, Table S1: The average and standard deviation of quality of life measurement, Table S2: The average and standard deviation of the Beck depression scale measurement, Table S3: Tests of within-subject effects (Beck’s depression scale, BDS), Table S4: The average and standard deviation of pain severity assessment using the NRS scale, Table S5: Tests of within-subject effects (NRS Scale), Table S6: McNemar’s test for “yes” vs “no” responses to the question about the participation in a walk in the last week, Table S7: McNemar’s test of whether the frequency of “yes” versus “no” responses to the question about spending most of the time in bed or in a chair in the past 7 days differed between measurements, Table S8: McNemar’s test of whether the frequency of “yes” versus “no” answers to the question about the patient’s perception of improvement in his or her own performance differed in subsequent measurements, Material M1: Original questionnaire used for the research, Material M2: Pre-validation of the questionnaire, Material M3: Original questionnaire used for the final evaluation of the program.

## References

[1] (2012). WHO. The World Health Organization Quality of Life (WHOQOL).

[2] Panzini R.G., Mosqueiro B.P., Zimpel R.R., Bandeira D.R., Rocha N.S., Fleck M.P. (2017). Quality-of-life and spirituality. Int. Rev. Psychiatry.

[3] Van Loon M.S., Van Leeuwen K.M., Ostelo R.W.J.G., Bosmans J.E., Widdershoven G.A.M. (2017). Quality of life in a broader perspective: Does ASCOT reflect the capability approach?. Qual. Life Res..

[4] Counted V., Possamai A., Meade T. (2018). Relational spirituality and quality of life 2007 to 2017: An integrative research review. Heal. Qual. Life Outcomes.

[5] De Walden-Gałuszko K., Ciałkowska-Rysz A. (2015). Medycyna Paliatywna.

[6] Ciałkowska-Rysz A., Dzierżanowski T. (2019). Medycyna Paliatywna.

[7] Harden K., Price D., Duffy E., Galunas L., Rodgers C. (2017). Palliative Care: Improving Nursing Knowledge, Attitudes, and Behaviors. Clin. J. Oncol. Nurs..

[8] WHO Definition of Palliative Care. http://www.who.int/cancer/palliative/definition/en/.

[9] Drop B., Barańska A., Firlej E., Janiszewska M., Jędrych M. (2017). Palliative care and the growing health needs of older people in Poland. J. Educ. Health Sport.

[10] IASP Terminology. https://www.iasp-pain.org/Education/Content.aspx?ItemNumber=1698#Pain.

[11] Mehta A., Chan L.S. (2008). Understanding of the Concept of "Total Pain". J. Hosp. Palliat. Nurs..

[12] Langley P., Müller-Schwefe G., Nicolaou A., Liedgens H., Pergolizzi J., Varrassi G. (2010). The societal impact of pain in the European Union: Health-related quality of life and healthcare resource utilization. J. Med. Econ..

[13] Paterniani A., Sperati F., Esposito G., Cognetti G., Pulimeno A.M.L., Rocco G., Diamanti P., Bertini L., Baldeschi G.C., Varrassi G. (2020). Quality of life and disability of chronic non-cancer pain in adults patients attending pain clinics: A prospective, multicenter, observational study. Appl. Nurs. Res..

[14] Varrassi G., De Conno F., Orsi L., Puntillo F., Sotgiu G., Zeppetella J., Zucco F. (2020). Cancer Pain Management: An Italian Delphi Survey from the Rational Use of Analgesics (RUA) Group. J. Pain Res..

[15] Antonucci L.A., Taurino A., Laera D., Taurisano P., Losole J., Lutricuso S., Abbatantuono C., Giglio M., De Caro M.F., Varrassi G. (2020). An Ensemble of Psychological and Physical Health Indices Discriminates Between Individuals with Chronic Pain and Healthy Controls with High Reliability: A Machine Learning Study. Pain Ther..

[16] Liampas A., Rekatsina M., Vadalouca A., Paladini A., Varrassi G., Zis P. (2020). Non-Pharmacological Management of Painful Peripheral Neuropathies: A Systematic Review. Adv. Ther..

[17] Zis P., Varrassi G., Vadalouka A., Paladini A. (2019). Psychological Aspects and Quality of Life in Chronic Pain. Pain Res. Manag..

[18] Miniszewska J., Chrystowska-Jabłońska B. (2002). Strategia radzenia sobie z chorobą nowotworową a jakość życia. Psychoonkologia.

[19] Tabassum S., Sheetal K., Joginder Y., Shalu T., Khumanshi Y., Pinky T. (2018). Role and importance of Physiotherapy during Palliative Care in India: A Review. World J. Res. Rev..

[20] McTiernan A., Friedenreich C.M., Katzmarzyk P.T., Powell K.E., Macko R., Buchner D., Pescatello L.S., Bloodgood B., Tennant B., Vaux-Bjerke A. (2019). Physical activity in cancer prevention and survival: A systematic review. Med. Sci. Sports Exerc..

[21] Bernhardt J., Dewey H., Thrift A., Donnan G. (2004). Inactive and alone: Physical activity within the first 14 days of acute stroke unit care. Stroke.

[22] López-Sendín N., Alburquerque-Sendín F., Cleland J.A., Fernández-de-las-Peñas C. (2012). Effects of Physical Therapy on Pain and Mood in Patients with Terminal Cancer: A Pilot Randomized Clinical Trial. J. Altern. Complement. Med..

[23] Pyszora A., Budzyński J., Wójcik A., Prokop A., Krajnik M. (2017). Physiotherapy programme reduces fatigue in patients with advanced cancer receiving palliative care: Randomized controlled trial. Support. Care Cancer.

[24] McGrillen K., McCorry N.K. (2014). A physical exercise programme for palliative care patients in a clinical setting: Observations and preliminary findings. Prog. Palliat. Care.

