# Supplementary material for: Influence of Physical Activity on Pain, Depression and Quality of Life of Patients in Palliative Care: A Proof-of-Concept Study
Source: J Clin Med. 2021 Mar 2;10(5):1012. doi: 10.3390/jcm10051012 (PMC7958598; doi:10.3390/jcm10051012)
Supplement: Supplementary file 1 [file jcm-10-01012-s001.pdf]

**Table S1. The average and standard deviation of quality of life measurement.**

| Quality of life measure: | Average | Mean standard error | Deviation | 95% confidence interval | |
|---|---|---|---|---|---|
| | | | | Lower limit | Upper limit |
| 1 | 2,543 | ,151 | 1,44810 | 2,244 | 2,843 |
| 4 | 3,272 | ,147 | 1,40723 | 2,980 | 3,563 |

*The average level of living quality measured by Likert scale 1-5.

**Table S2. The average and standard deviation of the Beck Depression Scale measurement.**

| **Meas-ure** | **Mean** | **Mean standard error** | **Deviation** | **95% confidence interval** | |
|---|---|---|---|---|---|
| | | | | Lower limit | Upper limit |
| 0 | 18,478 | ,532 | 5,10436 | 17,421 | 19,535 |
| 1 | 17,228 | ,551 | 5,28548 | 16,134 | 18,323 |
| 2 | 16,500 | ,560 | 5,37086 | 15,388 | 17,612 |
| 3 | 16,000 | ,572 | 5,48925 | 14,863 | 17,137 |
| 4 | 15,652 | ,569 | 5,45599 | 14,522 | 16,782 |

**Table S3. Tests of Within-Subjects Effects (Beck's depression scale - BDS).**

| | **Source** | **Type III of the sum of squares** | **df** | **Mean square** | **F** | **Rele-vance** | **Partial Eta square** |
|---|---|---|---|---|---|---|---|
| BDS | Sphericity Assumed | 464.000 | 4 | 116.000 | 140.559 | **.000** | 464.000 |
| | Greenhouse-Geisser | 464.000 | 1.666 | 278.483 | 140.559 | **.000** | 464.000 |
| | Huynh-Feldt | 464.000 | 1.694 | 273.987 | 140.559 | **.000** | 464.000 |
| | Lower-bound ep-silon | 464.000 | 1.000 | 464.000 | 140.559 | **.000** | 464.000 |
| Error (BDS) | Sphericity Assumed | 300.400 | 364 | .825 | | | 300.400 |
| | Greenhouse-Geisser | 300.400 | 151.621 | 1.981 | | | 300.400 |
| | Huynh-Feldt | 300.400 | 154.109 | 1.949 | | | 300.400 |
| | Lower-bound ep-silon | 300.400 | 91.000 | 3.301 | | | 300.400 |

**Table S4. The average and standard deviation of pain severity assessment using the NRS scale.**

| NRS measures | Average | Mean standard error | Deviation | 95% confidence interval | |
|---|---|---|---|---|---|
| | | | | Lower limit | Upper limit |
| NRS 0 | 4,033 | ,259 | 2,48711 | 3,518 | 4,548 |
| NRS 1 | 3,489 | ,219 | 2,09916 | 3,054 | 3,924 |
| NRS 2 | 3,207 | ,216 | 2,07305 | 2,777 | 3,636 |
| NRS 3 | 3,033 | ,205 | 1,96369 | 2,626 | 3,439 |
| NRS 4 | 2,674 | ,183 | 1,75480 | 2,311 | 3,037 |

**Table S5. Tests of Within-Subjects Effects (NRS Scale).**

| Source | | Type III of the sum of squares | df | Mean square | F | Rele-vance | Partial Eta square |
|---|---|---|---|---|---|---|---|
| NRS measures | Sphericity Assumed | 96.035 | 4 | 24.009 | 58.587 | **.000** | .392 |
| | Greenhouse-Geisser | 96.035 | 2.272 | 42.276 | 58.587 | **.000** | .392 |
| | Huynh-Feldt | 96.035 | 2.333 | 41.166 | 58.587 | **.000** | .392 |
| | Lower-bound epsi-lon | 96.035 | 1.000 | 96.035 | 58.587 | **.000** | .392 |
| Error (NRS measures) | Sphericity Assumed | 149.165 | 364 | .410 | | | |
| | Greenhouse-Geisser | 149.165 | 206.718 | .722 | | | |
| | Huynh-Feldt | 149.165 | 212.289 | .703 | | | |
| | Lower-bound epsi-lon | 149.165 | 91.000 | 1.639 | | | |

**Table S6.** McNemar's test for "yes" vs "no" responses to the question about the participation in a walk in the last week

| | $□^{□}_{(Yates)}$ | *p* | $p_{adj.}$ |
|---|---|---|---|
| survey 0 vs survey 1 | 68,94 | <0,001 | <0,001 |
| survey 0 vs survey 2 | 68,94 | <0,001 | <0,001 |
| survey 0 vs survey 3 | 55,13 | <0,001 | <0,001 |

| | | | |
|---|---|---|---|
| survey 0 vs survey 4 | 48,25 | <0,001 | <0,001 |
| survey 1 vs survey 2 | 54,79 | <0,001 | <0,001 |
| survey 1 vs survey 3 | 41,65 | <0,001 | <0,001 |
| survey 1 vs survey 4 | 35,21 | <0,001 | <0,001 |
| survey 2 vs survey 3 | 41,65 | <0,001 | <0,001 |
| survey 2 vs survey 4 | 35,21 | <0,001 | <0,001 |
| survey 3 vs survey 4 | 15,25 | <0,001 | 0,001 |

Table S7. McNemar's test of whether the frequency of "yes" versus "no" responses to the question about spending most of the time in bed or in a chair in the past 7 days differed between measurements

| | $\chi^2_{(Yates)}$ | $p$ | $p_{adj.}$ |
|---|---|---|---|
| survey 0 vs survey 1 | 66,95 | <0,001 | <0,001 |
| survey 0 vs survey 2 | 60,24 | <0,001 | <0,001 |
| survey 0 vs survey 3 | 17,59 | <0,001 | <0,001 |
| survey 0 vs survey 4 | 13,60 | <0,001 | 0,002 |
| survey 1 vs survey 2 | 54,57 | <0,001 | <0,001 |
| survey 1 vs survey 3 | 14,11 | <0,001 | 0,002 |
| survey 1 vs survey 4 | 10,53 | 0,001 | 0,012 |
| survey 2 vs survey 3 | 12,47 | <0,001 | 0,004 |
| survey 2 vs survey 4 | 9,10 | 0,003 | 0,026 |
| survey 3 vs survey 4 | 0,16 | 0,685 | 1,000 |

$\chi^2_{(Yates)}$ – wynik testu McNemara z poprawką Yatesa, $p$ – istotność statystyczna, $p$ – istotność statystyczna z korektą Bonferroniego

Table S8. McNemar's test of whether the frequency of "yes" versus "no" answers to the question about the patient's perception of improvement in his or her own performance differed in subsequent measurements

| | $\chi^2_{(Yates)}$ | $p$ | $p_{adj.}$ |
|---|---|---|---|
| survey 0 vs survey 1 | 58,37 | <0,001 | <0,001 |
| survey 0 vs survey 2 | 52,40 | <0,001 | <0,001 |
| survey 0 vs survey 3 | 42,48 | <0,001 | <0,001 |
| survey 0 vs survey 4 | 24,74 | <0,001 | <0,001 |
| survey 1 vs survey 2 | 12,66 | <0,001 | 0,004 |
| survey 1 vs survey 3 | 6,96 | 0,008 | 0,083 |
| survey 1 vs survey 4 | 0,43 | 0,511 | 1,000 |
| survey 2 vs survey 3 | 3,52 | 0,060 | 0,605 |
| survey 2 vs survey 4 | 0,00 | 1,000 | 1,000 |

| | | | |
|---|---|---|---|
| survey 3 vs survey 4 | 1,09 | 0,295 | 1,000 |

**Supplemental material #1**
Original questionnaire used for the research

Szanowni Państwo,

Uprzejmie prosimy o wypełnienie poniższego kwestionariusza będącego składową programu dydaktycznego mającego na celu edukację pacjentów oraz ich opiekunów w zakresie samodzielności oraz aktywności ruchowej chorych w aspekcie poprawy jakości życia
i zmniejszenia natężenia bólu całkowitego. Dane zebrane w badaniu zostaną zanonimizowane, a wyniki zostaną wykorzystane do celów naukowych.

Z góry dziękujemy za udział w badaniu.

1. PŁEĆ: K □ M □
2. WIEK: ……
3. WZROST: ……
4. WAGA: ……
5. CZY W OSTATNIM TYGODNIU CZUŁ PAN/PANI OGRANICZENIA W WYKONYWANIU CZYNNOŚCI DNIA CODZIENNEGO?

   □ NIGDY □ CZASAMI □ CZĘSTO □ BARDZO CZĘSTO
6. CZY W OSTATNIM TYGODNIU ODBYŁ PAN/PANI SPACER?

   □ TAK □ NIE
7. CZY W OSTATNIM TYGODNIU POTRZEBOWAŁ PAN/PANI POMOCY PODCZAS WYKONYWANIA CZYNNOŚCI HIGIENICZNYCH DNIA CODZIENNEGO?

   □ NIGDY □ CZASAMI □ CZĘSTO □ BARDZO CZĘSTO
8. CZY W CIĄGU OSTATNICH 7 DNI ZE WZGLĘDU NA ZŁE SAMOPOCZUCIE SPĘDZIŁ PAN/PANI WIĘKSZOŚĆ CZASU W ŁÓŻKU LUB FOTELU?

   □ TAK □ NIE
9. CZY W CIĄGU OSTATNICH 7 DNI WYMAGAŁ PAN/PANI POMOCY PODCZAS SPOŻYWANIA POSIŁKÓW?

   □ NIGDY □ CZASAMI □ CZĘSTO □ BARDZO CZĘSTO
10. CZY W CIĄGU OSTATNIEGO TYGODNIA ZAUWAŻYŁ PAN/PANI POPRAWĘ SWOJEJ SPRAWNOŚCI?

    □ TAK □ NIE

11. PROSZĘ OCENIĆ SWÓJ BÓL W SKALI OD 0 DO 10, GDZIE 0 OZNACZA CAŁKOWITY BRAK BÓLU, NATOMIAST 10 NAJGORSZY WYOBRAŻALNY BÓL.

 0  1  2  3  4  5  6  7  8  9  10

12. JAK OCENIA PAN/PANI SWOJĄ JAKOŚĆ ŻYCIA?

 BARDZO DOBRZE
 DOBRZE
 TRUDNO POWIEDZIEĆ
 ŹLE
 BARDZO ŹLE

<u>INFORMACJE WYPEŁNIANE PRZEZ OSOBĘ NADZORUJĄCĄ BADANIE</u>

1. CHOROBA PODSTAWOWA ZGODA Z KATALOGIEM NFZ KLASYFIKUJĄCA DO LECZENIA W PORADNI MEDYCYNA PALIATYWNEJ LUB HOSPICJUM DOMOWEGO:

   ……………………………….

2. WYNIK BMI:

   ………………………………

3. OCENA STANU SPRAWNOŚCI RUCHOWEJ WEDŁUG INDEKSU KARNOFSKY’EGO:

   ………………………………

4. LICZBA PUNKTÓW UZYSKANYCH W TEŚCIE DEPRESJI BECK’A:

   ……………………………….

NUMER BADANIA: ……………………

IDENTYFIAKTOR PACJENTA: …………

Supplementary material #2

## Pre-validation of the questionnaire

In order to validate the research tool used, the internal consistency of the two indicators was tested.
1. The first is related to physical fitness (items 6, 8 and 10),
2. The second is related to daily activities (items 5, 7 and 9).
For this purpose, the Kuder-Richardson (KR) coefficient 20 (for questions with a dichotomous response scale) and Cronbach's alpha (for questions with a simplified Likert scale) were used. The results of the analysis show a high internal consistency of the tool used in each of the five measurements. This means that the research tool used can be considered reliable.

Table 1. (in the reply to reviewer #3)
Reliability coefficients for two dimensions in five measurements, in which the constructed research tool was used

| | KR-20 | Cronbach's Alpha |
|---|---|---|
| survey 0 | 0,78 | 0,89 |
| survey 1 | 0,68 | 0,91 |
| survey 2 | 0,83 | 0,78 |
| survey 3 | 0,79 | 0,79 |
| survey 4 | 0,82 | 0,81 |

Supplemental material #3

## Original questionnaire used for the final evaluation of the program (Fig 11 in the text)

ANKIETA SATYSFAKCJI PACJENTA BIORĄCEGO UDZIAŁ W PROGRAMIE EDUKACYJNYM

Szanowni Państwo,

Mając na uwadze dobro naszych pacjentów, pragniemy poznać Państwa zdanie na temat wprowadzonego przez Nas programu dydaktycznego mającego na celu edukację pacjentów
oraz ich rodzin w zakresie samodzielności oraz aktywności ruchowej chorych w aspekcie poprawy jakości życia i zmniejszenia natężenia bólu wszechogarniającego.

Wszelkie sugestie oraz uwagi będą dla nas bardzo cenne i posłużą do korygowania niedociągnięć i podniesienia standardu oferowanych usług.

1. PŁEĆ

   □Kobieta □Mężczyzna
2. WIEK

   □18-30 lat □31-50 lat□51-70 lat □powyżej 71 lat
3. PRACA LEKARZA: JAK OCENIA PAN/PANI UPRZEJMOŚĆ, OKAZYWANIE ZROZUMIENIA I ZAINTERESOWANIA PRZEZ LEKARZY?

   □Bardzo dobrze □Dobrze □Trudno powiedzieć□Źle □Bardzo źle
4. PRACA LEKARZA: JAK OCENIA PAN/PANI JAKOŚĆ INFORMACJI PRZEKAZAYWANYCH PRZEZ LEKARZY PODCZAS SPOTKAŃ EDUKACYJNYCH

   □Bardzo dobrze □Dobrze □Trudno powiedzieć□Źle □Bardzo źle
5. PRACA PERSONELU PIELĘGNIARSKIEGO: JAK OCENIA PAN/PANI UPRZEJMOŚĆ, OKAZYWANIE ZROZUMIENIA I ZAINTERESOWANIA PRZEZ PIELĘGNIARKI?

   □Bardzo dobrze □Dobrze □Trudno powiedzieć□Źle □Bardzo źle
6. PRACA PERSONELU PIELĘGNIARSKIEGO: JAK OCENIA PAN/PANI JAKOŚĆ INFORMACJI PRZEKAZAYWANYCH PRZEZ PIELĘGNIARKKI PODCZAS SPOTKAŃ EDUKACYJNYCH?

   □Bardzo dobrze □Dobrze □Trudno powiedzieć□Źle □Bardzo źle
7. PRACA FIZJOTERAPEUTY: JAK OCENIA PAN/PANI UPRZEJMOŚĆ, OKAZYWANIE ZROZUMIENIA I ZAINTERESOWANIA PRZEZ FIZJOTERAPEUTĘ?

   □Bardzo dobrze □Dobrze □Trudno powiedzieć□Źle □Bardzo źle
8. PRACA FIZJOTERAPEUTY: JAK OCENIA PAN/PANI JAKOŚĆ INFORMACJI PRZEKAZAYWANYCH PRZEZ FIZJOTERAPEUTĘ PODCZAS SPOTKAŃ EDUKACYJNYCH?

   □Bardzo dobrze □Dobrze □Trudno powiedzieć□Źle □Bardzo źle
9. PRACA PSYCHOLOGA: JAK OCENIA PAN/PANI UPRZEJMOŚĆ, OKAZYWANIE ZROZUMIENIA I ZAINTERESOWANIA PRZEZ PSYCHOLOGA?

   □Bardzo dobrze □Dobrze □Trudno powiedzieć□Źle □Bardzo źle

10. PRACA PSYCHOLOGA: JAK OCENIA PAN/PANI JAKOŚĆ INFORMACJI PRZEKAZAYWANYCH PRZEZ PSYCHOLOGA PODCZAS SPOTKAŃ EDUKACYJNYCH?

    □Bardzo dobrze □Dobrze □Trudno powiedzieć□Źle □Bardzo źle

11. KTÓRE Z DZIAŁAŃ REALIZOWANYCH PRZEZ PROGRAM UZNAJESZ ZA NAJBARDZIEJ POTRZEBNE?

………………………………………………………………………………………………………
………………………………………………………………………………………………………

……………………………………………………………………………………………………

12. KTÓRE Z DZIAŁAŃ REALIZOWANYCH PRZEZ PROGRAM UZNAJESZ ZA ZBĘDNE?

……………………………………………………………………………………………………
……………………………………………………………………………………………………
……………………………………………………………………………………………………

13. JAKIE DZIAŁANIA UWAŻASZ, ŻE WARTO BYŁOBY WŁĄCZYĆ W DZIAŁALNOŚĆ PROGRAMU?

……………………………………………………………………………………………………
……………………………………………………………………………………………………
……………………………………………………………………………………………………

14. JAK CAŁOŚCIOWO OCECNIASZ WPROWADZONY PRZEZ NAS PROGRAM EDUKACYJNY?

□Bardzo dobrze □Dobrze □Trudno powiedzieć □Źle □Bardzo źle

Serdecznie dziękujemy za wypełnienie ankiety
